# Fas deficiency attenuates bone loss during antigen induced arthritis in mice

**DOI:** 10.1186/ar3639

**Published:** 2012-02-09

**Authors:** Elvira Lazic Mosler, Sania Kuzmac, Sanja Ivcevic, Danka Grcevic, Ana Marusic, Natasa Kovacic

**Affiliations:** 1 Laboratory for Molecular Immunology, University of Zagreb School of Medicine, Zagreb HR-10000, Croatia; 2 Department of Anatomy, University of Zagreb School of Medicine, Zagreb HR-10000, Croatia; 3 Department of Physiology and Immunology, University of Zagreb School of Medicine, Zagreb HR-10000, Croatia; 4 Department of Research in Biomedicine and Health, University of Split School of Medicine, Split HR-21000, Croatia

## Background

Antigen induced arthritis (AIA) is an experimental model of rheumatoid arthritis induced by methylated bovine serum albumin (mBSA) [1]. Hyperplastic synovia in AIA contains fibroblast-like synoviocytes (FLS) with reduced ability to differentiate into osteoblasts, chondroblasts or adipocytes [2]. Since Fas is shown to inhibit osteoblast differentiation [3], we were interested whether such inhibitory effect may contribute to the pathogenesis of AIA.

## Materials and methods

AIA was induced in mice with a Fas gene knockout (Fas -/-). Three weeks after pre-immunization with mBSA in complete Freund's adjuvant, wild-type (C57BL/6, wt) and Fas -/- mice were injected with mBSA into each knee, whereas controls were injected with equal volume of phosphate buffered saline (PBS). Three weeks after injection we assessed joint diameters, histology, μCT scans, and differentiation of bone marrow- and synovia-derived osteoblasts.

## Results

Knee diameters were increased in mBSA-injected wt mice compared to PBS-injected controls (3.21 ± 0.2 vs. 2.98 ± 0.1, p < 0.05, t-test), and this increase was not significant in Fas -/- mice (2.97 ± 0.2 vs. 2.87 ± 0.1). Histology revealed presence of synovial hyperplasia in both mBSA-injected groups, but mBSA-injected wt mice had decreased trabecular bone volume in distal femoral metaphyses (BV/TV) compared to controls (1.08 ± 0.57 vs. 2.55 ± 0.43; p < 0.05, t-test). There was no significant difference between mBSA-injected and control group in Fas -/- mice (2.34 ± 0.62 vs. 2.61 ± 0.65). μCT analysis showed that mBSA-injected wt mice had decreased BV/TV (2.99 ± 0.19 v. 1.96 ± 0.19; p < 0.001, t-test) and trabecular number (TbN) (1.03 ± 0.03 vs. 0.64 ± 0.02), as well as increased trabecular separation (TbSep) (256,89 ± 1395,12 vs. 312.40 ± 1323.91), compared to controls. mBSA injected Fas -/- mice had decreased TbN compared to controls (0.815 ± 0.01 vs. 0.64 ± 0.04; p < 0.05, t-test), with no significant difference in other trabecular parameters. Osteoblast differentiation was increased in both wt and Fas -/- mBSA-injected mice.

## Conclusions

Our study demonstrated that Fas deficiency attenuated the development of clinical signs and bone loss in AIA. The mechanisms of this phenomenon need to be clarified.

## References

[B1] van den Berg WB Murine antigen-induced arthritis Methods in Molecular Medicine, Volume 136: Arthritis Research 2007 2 Cope AP Totowa (NJ): Humana Press Inc 243 253 10.1007/978-1-59745-402-5_18 17983153

[B2] Li X Makarov SS An essential role of NF-kappaB in the "tumor-like" phenotype of arthritic synoviocytes Proc Natl Acad Sci USA 2006 103 17432 7 10.1073/pnas.0607939103 17088540 PMC1859946

[B3] Kovacic N Lukic IK Grcevic D Katavic V Croucher P Marusic A The Fas/Fas-ligand system inhibits differentiation of murine osteoblasts but has a limited role in osteoblast and osteoclast apoptosis J Immunol 2007 178 3379 89 17339432 10.4049/jimmunol.178.6.3379 PMC2774560

